# The Literary Estimate of Medical Art

**Published:** 1877-07

**Authors:** 


### ARTICLE VI.

### *The Literary Estimate of Medical Art.*

It requires but a slight tincture of literature to notice how physicians have, as a class, gained in social standing since the revival of literature, and especially since the birth of scientific observation, properly so called.

Take as an example of the sixteenth century, Montaigne, of the seventeenth Moliere, and of the eighteenth Lesage, all names of the highest note in literature, and compare their frequent descriptions of medical men and medical art with those in the current literature of this century, and how striking the contrast!

In that admirable and characteristic one of Montaigne's essays "On the Resemblance of Children to their parents,"

he gives vent to all his scorn and contempt for physicians. He ransacks antiquity for anecdotes to cast contumely upon them. He quotes with zest the reply of Nicoles to a physician who boasted of the greatness of his art: "Yes," replied the Greek, "that's so, it gives you the privilege of murdering with impunity." He cites with pleasure the law of the Egyptians, who made the doctor responsible for the life of the patient, if he was not cured in three days. He asserts that Cato, the Censor, did a meritorius act in banishing all the physicians from Rome (which Cato never did by the by.) All this and much more he brings in as germane to the subject of his chapter, by introducing an anecdote of his old uncle, who, when he was told that the doctor had been sent for, threw himself back in despair and exclaimed, "I am done for, then."

The doctor of medicine was the favorite butt of Moliere's wit. The "faculty" is represented as a compound of ignorance, pedantry and intolerance; their consultations are jealous quarrels or bragging speeches; their great anxiety that their rivals shall not succeed! One of them says: "Since heaven has made the people so infatuated with us, do not let us open their eyes by our quarrels, but rather profit by their silliness." Another tells the father of a sick young lady, "if your daughter does die, you will have the consolation of knowing she died according to proper forms. Better to die according to rule than recover against rule."

Lesage, in the famous picture of Dr. Sangrado, has left an immortal satire on the profession; but it is but one of many passages in his works where he heaps his ridicule on our avocation.

But see how great a change takes place after the commencement of scientific thought in the latter half of the last century.

The great GOETHE was one of the first to recognize it. In his beautiful autobiographical work, "Truth and Poetry from my Life," he tells us—

"At Strasbourg, the greater part of my associates at meals were medical students. These, as is known, are the only students who instruct themselves with energy concerning their science and profession, beyond the usual hours of study. This lies in the nature of the case. The objects of their labors require the greatest thought and are of the highest kind ; they are at once the most simple and the most complex. Medicine concerns the whole human race, because the whole human race are concerned with it. All that the youth learns points directly to a weighty, indeed dangerous, but in many senses a remunerative profession. He throws himself, therefore, with passion, to find out what is to learn and what to practice, in part because the things themselves interest him, and in part because of the pleasant prospect of independence and riches which is opened to him."

Elsewhere he tells us that there are but two vocations the pursuers of which are received as intimates and equals by those of the loftiest birth and most exalted positions, the ministry and medicine, because, he adds, at some period in every life the devoted services of these two are imperative and priceless.

So we could quote fromBalzac, from Dickens, Thackeray, from hosts of novelists and dramatists of lesser note, in whose pages similar tributes are paid to the importance and to the real present worth of the medical art of this century. Indeed, the writer who would dare to indulge now in anything like the contemptuous expressions of Montaigne would very affectually write himself down an ass, for there is too much of positive knowledge and efficient remedial resources in the medical art of to-day for any one to contemn it, or for any man of sense to dispense with it.—*Editorial in Med. and Surg. Reporter.*

---

